# Ultrasonographic Detected Adrenomegaly in Clinically Ill Cats: A Retrospective Study

**DOI:** 10.3390/vetsci9080420

**Published:** 2022-08-09

**Authors:** João Oliveira, Maria Joana Dias, Ana Paula Fontes, Ryane E. Englar, Gonçalo Vicente, Rui Lemos Ferreira, Sara Galac, Rodolfo Oliveira Leal

**Affiliations:** 1 Veterinary Teaching Hospital, Faculty of Veterinary Medicine, University of Lisbon, 1300-477 Lisbon, Portugal; 2 CIISA—Centre for Interdisciplinary Research in Animal Health, Faculty of Veterinary Medicine, University of Lisbon, 1300-477 Lisbon, Portugal; 3 Associate Laboratory for Animal and Veterinary Sciences (AL4AnimalS), 1300-477 Lisbon, Portugal; 4 School of Healthcare, University of Algarve, 8005-139 Faro, Portugal; 5 College of Veterinary Medicine, University of Arizona, 1580 E Hanley Blvd., Oro Valley, AZ 85737, USA; 6 Department of Clinical Sciences, Faculty of Veterinary Medicine, Utrecht University, Yalelaan 1, 3584 Utrecht, The Netherlands

**Keywords:** adrenomegaly, ultrasound, cats, clinically ill, endocrine

## Abstract

**Simple Summary:**

Scientific literature regarding adrenomegaly in cats is scarce. This study aimed to characterize feline adrenomegaly, estimating its prevalence among clinically ill cats, and assessing whether it was suspected or an incidental finding. Abdominal ultrasonography reports of cats presenting to a veterinary teaching hospital over 28 months were reviewed. Cats showing adrenomegaly (defined as one or both adrenal glands having a dorsoventral axis >4.8 mm) were selected. Medical records and adrenal ultrasonographic findings were detailed. From a total of 983 ultrasonographical reports, 68 (7%) disclosed adrenomegaly. European/domestic short-hair male castrated cats were overrepresented. Adrenomegaly was an incidental finding in most cats (62/68; 91%), while in the remaining, it was identified in the context of exploration of a potential adrenal disease. Chronic kidney disease was the most frequent disease identified in clinically ill cats showing adrenomegaly (27/68; 37%), followed by endocrinopathies. Adrenomegaly was bilateral in more than half of cases, and, in unilateral ones, it was more prevalent on the left side, with a normal-sized contralateral adrenal gland. Comparing both adrenals, left adrenal demonstrated a larger size and a tendency to oval shape. This study assessed the prevalence of adrenomegaly in clinically ill cats, reinforcing it can be an incidental ultrasound finding.

**Abstract:**

This retrospective study aimed to assess the prevalence of ultrasonographic detected adrenomegaly in clinically ill cats, evaluating the final established diagnosis, describe adrenal ultrasound findings and if the adrenomegaly was suspected or incidental. Abdominal ultrasonography reports of cats presenting to a veterinary teaching hospital between October 2018 and February 2021 were retrospectively reviewed. Cats showing adrenomegaly (one or both glands having a dorsoventral axis >4.8 mm) were selected and medical records respectively evaluated. Nine-hundred and eighty-three ultrasonographical reports were selected, of which, 68 (7%) disclosed adrenomegaly. European/Domestic Short-Hair (62/68; 91%) male (44/68; 65%) castrated (35/44; 80%) cats were overrepresented. Adrenomegaly was an incidental finding in 62/68 (91%) cats while in 6/68 (9%) it was identified in the context of investigating a potential adrenal disease. Concerning established diagnosis, chronic kidney disease was overrepresented (25/68; 37%), followed by endocrinopathies (20/68; 29%). Adrenomegaly was bilateral in 53% (36/68) of cases. In unilateral cases (32/68; 47%), it was more prevalent on the left side (23/32; 72%), with a normal-sized contralateral adrenal gland. Left adrenal demonstrated a larger size and a tendency to oval shape. This study assesses the prevalence of adrenomegaly in clinically ill cats, reinforcing it can be an incidental ultrasound finding.

## 1. Introduction

Abdominal ultrasonography (AUS) is an important step in the diagnostic work-up for many feline diseases [1]. Although there are two studies that report the ultrasonographic (US) measurements of adrenal glands in cats with endocrine diseases [2,3], except cases of neoplasia, scientific literature regarding adrenomegaly in cats without a primary adrenal disease is scarce. Moreover, adrenomegaly can be an incidental finding and, when documented in cats without a primary adrenal disease, it has been attributed to chronic stress [4,5]. A publication by Zatelli et al. (2007) was the first of its kind to establish reference ranges for sizing adrenal glands when considering non-endocrine diseased cats [5]. A subsequent study confirmed that mean adrenal measurements are marginally higher in chronically ill cats as compared to healthy ones [4]. Authors inferred that adrenal gland enlargement in sick cats was due to the impact of disease (e.g., severity and chronicity) on the feline hypothalamic-pituitary-adrenal axis.

Next to stress of chronic illness, the differential diagnoses of adrenomegaly in cats include: adrenal cysts [6], hematomas [7,8], abscesses, granulomas [9,10,11], inflammation (adrenalitis) [7], hyperplasia (e.g., secondary to pituitary-dependent hypercortisolism, acromegaly or hyperthyroidism) [2,3,4,12,13,14,15,16,17], cortisol-secreting tumors [17,18], primary hyperaldosteronism [4,8,19,20,21], sex hormone-producing neoplasia [22,23,24], pheochromocytoma [25,26], malignant lymphoma [27], metastasis [28,29], and amyloidosis of the adrenal glands [30].

This study retrospectively revises cases of AUS detected adrenomegaly in clinically ill cats, aiming to characterize feline adrenomegaly, estimating its prevalence among clinically ill cats, and assessing whether it was suspected or an incidental finding.

## 2. Materials and Methods

A retrospective cross-sectional study was conducted in which all AUS reports of clinically ill cats presenting to the Veterinary Teaching Hospital—Faculty of Veterinary Medicine, University of Lisbon, between October 2018 and February 2021 were reviewed. Cats were included if they showed an AUS detected adrenomegaly, defined as one or both glands having a dorsoventral axis greater than 4.8 mm in longitudinal section [4,31]. Dorsoventral axis, either on sagittal or transverse section, has been proven to be more reliable and associated with less intra and inter-operator variability [32], reason why it was use for a more accurate assessment of adrenal size. In cats that underwent serial AUS, only findings from the first examination were analyzed to prevent duplication of data.

Each animal’s medical records were subsequently mined for data using practice management software (GuruVet^®^, Lendarius Agency, Santa Maria da Feira, Portugal). Data were compiled with regards to cat’s signalment, rationale(s) for diagnostic imaging, and final diagnosis (confirmed or suspected). Adrenomegaly was deemed relevant if differential diagnoses were considered and/or if specific add-on endocrine imaging and/or histopathological tests were performed or recommended by clinicians following AUS. Some of the cases underwent AUS because they were suspected to have adrenal disease. For others, adrenomegaly was considered an incidental finding. Cats were ultimately grouped as having an endocrinopathy or a non-endocrine disease.

**Figure 1:**
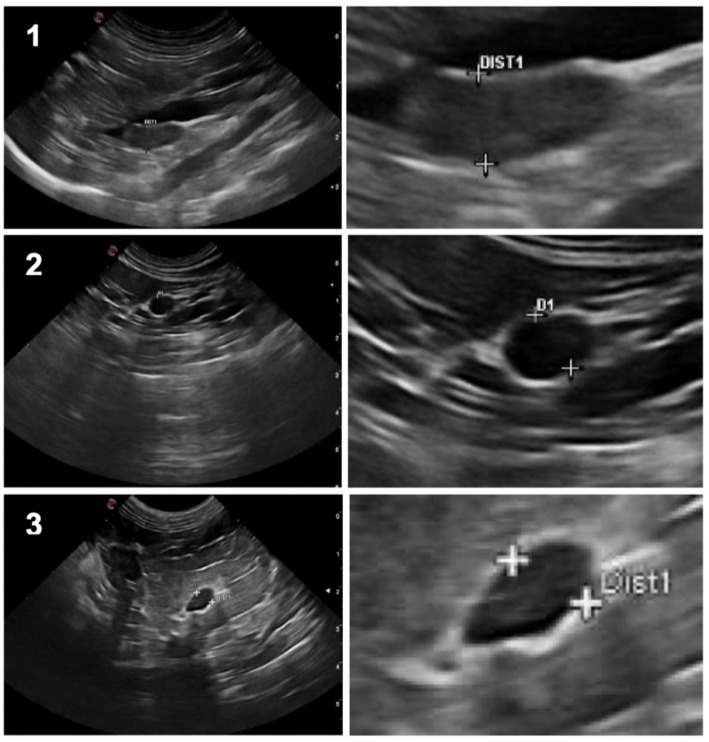
On the left column: Ultrasound still images illustrating the three different shapes of feline adrenal glands: 1. Bipolar; 2. Oval; 3. Fusiform; On the right column: respective “zoom-in” image, showing the dorsoventral axis measurement.

Stored adrenal US still images (Figure 1) were reviewed and interpreted by a certified-holder ultrasonographer (RLF), to identify whether adrenomegaly was unilateral or bilateral, and to evaluate adrenal dimension, limits, shape, cortico-medullary differentiation, echogenicity (assessed in a subjective comparison with its normal patterns), echotexture, hyperechogenic foci, and focal lesions. Although hyperechoic foci, in theory, may be considered focal lesions, for the purpose of this study, it refers to adrenal glands with normal echogenicity, structure, without nodular lesions but showing hyperechoic foci (an ultrasound finding which can occur mainly in old cats and without truly clinical significance), while focal lesions refer to adrenal glands with nodules.

Over the study period, stored images were obtained using four different ultrasound scanners (MyLabTMAlpha, Esaote^®^; MyLabTMOmega, Esaote^®^; MyLabTMX7VET, Esaote^®^; Vivid S6, GE Healthcare^®^). Cases in which US images were unavailable were only included if the dorsoventral axis of each adrenal gland was detailed in medical records.

The collected data were registered and organized in a database (Microsoft Excel Office 365^®^ 2019 software). For statistical analysis, a specific commercial software was used (IBM SPSS Statistics^®^ version 26). To determine the normality of the quantitative variables (age, weight and dimension of the left and right adrenals) the Kolmogorov–Smirnov test was applied. For normal distributed data, results were presented as mean ± standard deviation. For non-normal data, results were presented as median ± interquartile range. Data concerning age and weight were grouped according to cutoffs, set as ≥ or <10 years and ≥ or <4 kgs, respectively. Specifically concerning body weight, dimensions of the dorsoventral axis of right and left adrenals were compared between groups (≥ or <4 kgs), using non-parametric tests (Mann Whitney U test); for comparison of qualitative variables, a Chi-Square Test was used. For the echographic comparison between the left and right adrenal, the Paired T Test was performed in the variable “dimension” and the McNemar test for “limits”, “shape”, “cortico-medullary differentiation”, “echogenicity”, “echotexture”, “hyperechogenic foci”, and “focal lesions”. Statistical significance was set at <0.05 for a 95% confidence interval. Percentages were rounded to the nearest units, excepting values below 2% in which the decimal percentage is presented.

## 3. Results

A total of 983 AUS reports were evaluated and 68 cats met the inclusion criteria for adrenomegaly, representing a prevalence of 7%. Sixty-three (63/68; 93%) AUS were performed by the same operator (RLF). Concerning the remaining 915 cases, AUS were performed by the same ultrasonographist (RLF), and by another member of the same diagnostic imaging department, both certificate-holders in veterinary diagnostic imaging.

Sixty-two of the cats (62/68; 91%) were European/Domestic Short-Hair, four (4/68; 6%) Siamese and two (2/68; 3%) Persian cats. The mean age and weight were 11.6 ± 4.3 (range: 2–22) years and 4.0 kg ± 1.7 (range: 1.6–8.5), respectively. Most of the cats (44/68; 65%) were males, of which 80% (35/44) were castrated. Females represented 35% (24/68) of the sampled cats, of which the majority (19/24; 79%) had been spayed.

Proportions were compared within each parameter (breed, age, weight, gender, neuter status) and results are summarized in Table 1. European, castrated male cats and old ones were significantly overrepresented (*p* < 0.05). Concerning body weight, there was no significant difference between the proportion of cats weighting <4 kg or >4 kg showing adrenomegaly (*p* = 0.225). Detailing adrenal dimensions for each group, cats weighting <4 kg showed a median dorsoventral axis of 5.2 (±0.65) and 5.0 (±0.95) mm for left and right-adrenal, respectively. Cats weighting > 4 kg had 5.5 (±1.15) and 5.5 (±1.05) mm of median dorsoventral axis for left and right-adrenal, respectively. There were no significant differences on adrenal dimensions between these two groups (*p* = 0.49 and *p* = 0.06 for left and right adrenal dorsoventral axis, respectively).

**Figure 2:**
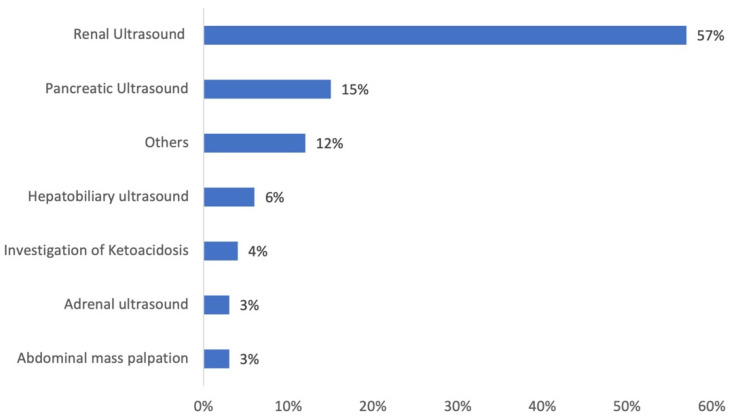
Main reasons for performing abdominal ultrasound in cats showing adrenomegaly. “Others” category consists of a percentage of 1.5% each, detailing: abdominal pain, lymph node investigation, uterine ultrasound, weight loss, suspected gastrointestinal foreign body, acute vomiting, chronic vomiting, and medical exploration of insulin resistance.

The rationales for imaging cats’ abdomens were obtained from the clinical records and are summarized in Figure 2. Renal US for the purpose of staging chronic kidney disease (CKD) was overrepresented as the primary reason to perform AUS (*p* < 0.001).

Adrenomegaly was an incidental finding in a total of 62/68 (91%) cats (*p* < 0.001), while in the remaining 6/68 (9%) it was identified in the context of exploration of a potential adrenal disease. Detailing, adrenomegaly was identified during AUS to work up cases of diabetic ketoacidosis (3/68; 4%), primary hyperaldosteronism (2/68; 3%), and insulin resistance (1/68; 2%).

Adrenomegaly was functionally unexplored in 56/68 cats (82%). In the remaining 12/68 (18%), it was found relevant for the medical investigation. However, despite endocrine tests were suggested, they were only performed in half of these cases: aldosterone and plasma renin activity were assessed in five cases (5/6; 83%) and a low-dose dexamethasone suppression test was performed in the remaining one (1/6; 17%).

**Figure 3:**
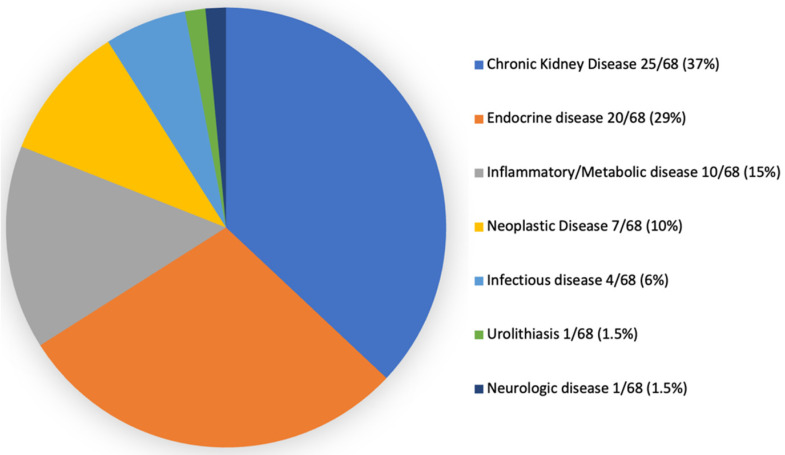
Final diagnosis in clinically ill cats noticed with adrenomegaly.

Regarding final diagnoses (Figure 3), CKD (25/68; 37%) was overrepresented. Endocrine disease was the second most frequent diagnostic group (20/68, 29%), including hyperthyroidism (9/68; 13%), diabetes mellitus (7/68; 10%), primary hyperaldosteronism (2/68, 3%), pituitary-dependent hypercortisolism (1/68, 1.5%), and hypersomatotropism (1/68, 1.5%). Inflammatory/metabolic disease accounted for 15% of cases (10/68), including pancreatitis (5/68, 7%), gastrointestinal foreign body (2/68, 3%), obstructive lower urinary tract disease (2/68; 3%), and lymphoplasmocytic duodenitis (1/68; 2%). Neoplasia was the final diagnosis in 10% of the cases (7/68) and included mammary neoplasia (2/68; 3%) and one case of each (1/68; 1.5%), detailing: cervical and base tail fibrosarcoma, left suprascapular fibrosarcoma, laryngeal lymphoma, alimentary lymphoma, and uterine adenocarcinoma. Infectious etiology was the final diagnosis for 6% (4/68) of cats, detailing: parasitic (toxoplasmosis—2/68, 3%) and bacterial (urinary tract infection—1/68, 1.5%; pyelonephritis—1/68, 1.5%). Urolithiasis and neurological disease (idiopathic epilepsy) accounted for a frequency of 1.5% (1/68) each.

Comparing the frequency of endocrine (20/68; 29%) versus non-endocrine cases (48/68; 71%), the later were over-represented (*p* < 0.001).

Adrenomegaly was bilateral in 36/68 (53%) of the cases, and unilateral with normal-sized contralateral adrenal gland in 32/68 (47%). There was no statistically significant difference in the prevalence of bilateral enlargement as opposed to unilateral (*p* = 0.628). Of the 32 unilateral cases, 23 (72%) were on the left and 9 (28%) on the right. This apparent difference in regional enlargement of the adrenals was statistically significant (*p* = 0.013).

Although all 68 cases had adrenal gland measurements within the medical records, stored images were only available for 64/68 cases (94%). Detailed findings are shown in Table 2.

Concerning left adrenal gland findings, it was mainly oval shaped with a mean dorsoventral axis (height) size of 5.5 ± 0.8 mm, with regular limits, without cortical-medullary differentiation, and without hyperechogenic foci and focal lesions. It demonstrated mixed echogenicity and homogeneous echotexture. By contrast, the right adrenal gland tended to take on a bipolar shape without corticomedullary differentiation. It had an average height of 5.1 ± 0.9 mm, with regular limits. The right adrenal gland was also hypoechogenic, homogeneous, and without hyperechogenic foci and focal lesions. The left adrenal gland tended to be larger (*p* = 0.005) and more ovoid in shape (*p* = 0.005) as compared to the right adrenal gland. However, despite the differences in dimension between left and right, the magnitude of this difference was small (Cohen’s d = 0.35).

## 4. Discussion

This study evaluated feline adrenomegaly detected on AUS, documenting a prevalence of 7% among clinically ill cats. It further characterized clinical findings in terms of whether adrenomegaly was unilateral or bilateral, incidental or suspected and potentially related to the current diagnosis of an endocrine or non-endocrine disease.

The obtained prevalence was higher compared to that of adrenal tumors, which have previously been described in the literature as representing 0.2% of feline neoplasms. This can be somehow explained because feline adrenomegaly is not necessarily associated with adrenal neoplasm [31].

Feline adrenomegaly was mainly detected in castrated European/Domestic Short-Hair cats and older than 10 years (mean 11.6 years old). As European/Domestic Short-Hair cats are very common in daily practice, it is unsurprising that this breed is overrepresented in this study. Considering neutering status, our study demonstrated adrenomegaly occurs more in neutered cats which agrees with a previous work of Combes et al. (2013) [4]. This maybe can be explained by the increase in the plasma aldosterone/renin activity ratio that is seen in neutered cats [33] or just because most of the hospital’s cat population were neutered. The fact that adrenomegaly is more prevalent in older animals may be related to some degree of aging/prone to diseases, which may contribute, albeit to a lesser degree, to gland enlargement [34,35].

In this study, a 4.8 mm cut-off of dorsoventral axis was defined for adrenomegaly. We have decided to use this upper-limit value, in agreement with a previous study on ultrasonographic appearance of adrenal glands in healthy and sick cats [4]. This study had a high number of US-measured adrenals from healthy and sick cats, having found that adrenal height was 3.0–4.8 mm (cranial dorso-ventral axis) and 3.0–4.5 mm (caudal dorso-ventral axis). The defined upper-limit in the present study also overlaps with the results obtained for healthy cats weighting >4–8 kg, from a recent study of Pérez-Lopéz et al. [36], in which dimensions were correlated with body weight. The present study focuses on sick cats, which can show adrenomegaly for an undefined period. Assuming weight can vary over time in chronic illness, and the registered body weight refers to the moment in which adrenomegaly was first identified, it does not necessarily reflect the “healthy” weight of the cat. We recognize that, by using a higher cut-off, we may have missed cases of adrenomegaly in cats weighting <4 kg, in case we would follow the healthy-cats cut-off, as defined by Pérez-Lopéz et al. [36]. Nonetheless, on the evaluation of sick-cats and given the paucity of studies on cut-off assessment, the upper-limit of 4.8 mm for dorsoventral axis seemed more reasonable to be used in the present study [4]. Using this cut-off, it was assessed whether adrenomegaly was more frequent in cats weighting <4 kg or >4 kg and results shown there was no difference on its prevalence among groups. Therefore, we could infer that body weight does not seem to be determinant on the assessment of adrenomegaly in clinically ill cats. Although this finding may be controversial, these results are in agreement with previous studies in which body weight, body surface, and body condition appear to have no effect on adrenal size [4,5]. Nonetheless, as documented by Pérez-López et al., weight should be considered when determining the dimensions of the adrenal glands of healthy cats [36].

Only in a minor percentage of cats was a precise intention to evaluate adrenal glands. This stresses that, except for the exploration of specific endocrine contexts such as insulin resistance, clinicians do not often perform AUS with the intent to explore adrenals in cats. This is in part justified by the fact that adrenal diseases seem to have a less common expression in cats when compared to dogs in which diseases such as hypercortisolism is far more common.

Regarding the final established diagnosis, some of them could be related or could have been the cause for the identified adrenomegaly; however, additional endocrine tests should have been done to rigorously exclude a primary adrenal disease.

Chronic kidney disease was overrepresented. The occurrence of adrenomegaly in cats with CKD is ambiguous as it can be both a cause of secondary hyperaldosteronism and/or a consequence of primary hyperaldosteronism. The renin-angiotensin-aldosterone system has been implicated in progressive renal sclerosis, with recent evidence indicating that not only angiotensin [34,35,37] but also aldosterone contributes to the progression of kidney damage by promoting thrombosis and fibrosis [38,39,40]. Thus, the occurrence of primary hyperaldosteronism can enhance the development of CKD. The development of secondary hyperaldosteronism occurs because of a compensatory mechanism to minimize the electrolyte losses of the disease secondary to polyuria. In the cases of CKD in this study, adrenomegaly was not characterized by the presence of nodules (except for one case), highlighting that secondary hyperaldosteronism seemed plausible. Nonetheless, primary hyperaldosteronism secondary to an adrenocortical hyperplasia cannot be excluded only based on these findings. Further exploration and prospective studies including aldosterone and renin activity measurement would be important to clarify cases of CKD with adrenomegaly in cats. This would make it possible to classify the type of hyperaldosteronism, and, in the case of primary cause, it would imply the institution of more appropriate therapeutic measures. It is worthwhile to mention that the two cases of primary hyperaldosteronism in this study were associated with the presence of adrenal nodules.

Hyperthyroidism was the second most prevalent disease in this study, standing out mainly as the most prevalent endocrine disease when adrenomegaly was detected. Hyperthyroidism is a well-documented differential for adrenomegaly and can be explained by the increase in circulating thyroid hormone that stimulates cortisol clearance and ACTH release, thus causing a secondary bilateral thickness of the adrenal cortex [2,4,41]. Hyperthyroid cats also exhibit an exaggerated response to minimal stress, which may be associated with an activation of the hypothalamic-pituitary-adrenal axis and an increase in the size of the adrenals [41].

Diabetes mellitus was the third most prevalent disease in this study, being the second most prevalent endocrine disease when adrenomegaly was detected. Considering the only study performed on the US dimensions of the adrenal glands in diabetic cats without hypercortisolism [42], it was concluded that diabetes mellitus does not cause an adrenal enlargement. However, in that study, the US evaluation conducted was performed at the early diagnosis, stressing that there may not have been enough time to induce adrenal changes.

Curiously, either hypercortisolism and hypersomatotropism, both well-known differentials of adrenomegaly in cats, were identified in a minor percentage of cases in the present study. However, not all cats were tested for those. It is commonly accepted that cases of adrenomegaly with pituitary-dependent hypercortisolism and hypersomatotropism are justified by cortical hyperplasia induced by ACTH and IGF-1 (Insulin-like Growth Factor 1), respectively [3,15]. The minor percentage observed in this study supports the rare frequency of these endocrine diseases in daily practice. However, as low-dose dexamethasone suppression test or IGF-1 dosage were not performed in all cats showing adrenomegaly, the prevalence of both diseases may be underestimated in the present study.

A smaller percentage of cases had pancreatitis as the final diagnosis when adrenomegaly is detected. Literature concerning adrenomegaly in pancreatitis is scarce, and it is not known, to date, whether there is a causal relationship between these conditions. In these cases, adrenomegaly was not further detailed, rendering difficult to investigate a potential correlation between these diseases [1].

Apart from CKD, pancreatitis, and the above-mentioned endocrine disorders, several other causes were reported as a suspected or final diagnosis in clinically ill cats with adrenomegaly. Due to the low expression of each disease, it would not be rigorous to speculate about the main causes of adrenomegaly. However, that seems plausible that regardless the final diagnosis, adrenomegaly can be justified by a metabolic adaptation to chronic stress/illness. Nevertheless, endocrine tests should have been done in all cases, to correctly exclude a primary adrenal disease. Concerning neoplastic disease, the possibility of adrenal metastases could be evoked although that would only be confirmed with cytology or histopathology, both not performed in any of the cases from this study.

Concerning laterality of adrenal enlargement, this study shows that cats can present in similar proportions a bilateral or unilateral enlargement and in all cases with unilateral enlargement, the contralateral adrenal was normal-sized.

The individual assessment of left and right adrenal gland allowed a better characterization of adrenomegaly in feline species. To the authors’ knowledge, there are no published studies on the ultrasound characterization of adrenomegaly in clinically ill cats, being these results the first data concerning left and right individual findings. When evaluating differences between right and left adrenal gland, it was shown that dimension and shape were significantly distinct. Comparing right and left adrenal size, this study showed that left adrenal is overall larger, although the difference was small. One possible justification is that left adrenal tends to show more structural changes than the right adrenal. In cats, the relationship between the left adrenal and the cranial pole of the left kidney is variable due to the latter’s greater mobility [43]. Thus, the question leads to speculate whether this greater mobility will not provide more space for the growth and shape change of the left adrenal. Although all the forms are represented in this study, the left adrenal was more often oval which is not in agreement with some previous studies supporting that bipolar was the most prevalent [2,4]. It is known that adrenal gland’s shape can vary in cats and is affected by the accuracy of the sagittal plane [1]. According to the slight obliqueness of the adrenal poles, the feline adrenal can present either a bipolar or a more oval shape which can in part affect the obtained results and justify the observed differences.

This study supports that adrenomegaly tends to be accepted in the medical-veterinary community as an US finding. Previous studies have documented that stress may affect adrenal gland size [4,5]. Being cats so prone to stress, it is discussable whether adrenomegaly can potentially be secondary to chronic stress/illness which invariably ends up as a pretext to almost never explore it. Further studies are needed to clearly understand the clinical relevance and to assess severity of adrenomegaly in healthy and sick cats.

Due to its retrospective nature, this study has several limitations. First, although the interpretation and evaluation of the US images were carried out by only one person, the technical procedure was performed by two people which can contribute to some subjectivity by decreasing the consistency of the US technique. Second, the use of four different sonography machines to gather data may have contributed to subtle variations in results. Data collection was difficult due to missing information from several cases. Moreover, the lack of endocrine and histopathological tests was one of the major limitations of this study. Functional characterization of the adrenals with endocrine tests and, ideally, histopathology of the adrenals would have been particularly useful because it would allow endocrine/functional and tissue characterization, permitting a definitive diagnosis of the cause of adrenomegaly. In this study, only a cause/effect attempt was considered. However, it cannot be fully proven without performing endocrine and histopathological tests. Its absence can be justified by the poor clinical value attributed to adrenomegaly in daily practice and by the possible owner’s financial commitment for further investigation.

Although this study opens new insights about US detected feline adrenomegaly, future prospective studies including cats with adrenomegaly submitted to endocrine exploration according to clinical relevance should be considered. Only with functional and eventually histopathological complementarity is it possible to correctly assess adrenomegaly and its clinical significance, correlating it with the US images and established diagnoses. It would also be interesting to perform kinetic studies on the time required for the adrenal gland to increase or decrease in size in relation to activation or inhibition of the hypothalamic-pituitary-adrenal axis, correlating with levels of cortisol, thyroxine, aldosterone and serum or urinary metanephrines. Since cats are particularly susceptible to stress, it would be important to clarify the impact of stress and chronic disease on the architecture of the adrenal glands by assessing whether adrenomegaly is a dynamic or static phenomenon over time.

## 5. Conclusions

Adrenomegaly was noticed in more cats without a suspected endocrinopathy than those with endocrinopathy, and feline adrenal enlargement was only considered relevant for the clinical diagnosis in few cases. According to this study, adrenomegaly has a prevalence of 7% in clinically ill cats and is mainly an US finding detected in European/Domestic Short-Hair castrated males, aged over 10 years and diagnosed with CKD. There is no significant difference between cases of bilateral and unilateral feline adrenomegaly. However, in unilateral cases, adrenomegaly is more prevalent on the left side and with normal-sized contralateral adrenal gland. Comparing both adrenals in the group of cats identified as having adrenomegaly, the left adrenal demonstrates a larger size and tends to be oval shape.

## Figures and Tables

**Table 1:** Signalment of clinically ill cats identified with adrenomegaly.

Characteristics	Classification	n = 68	*p* Value
Breed	Domestic Short-Hair	62 (91%)	<0.001
	Siamese	4 (6%)	
	Persian	2 (3%)	
Gender	Male	44 (65%)	0.015
	Female	24 (35%)	
Age ^1^	<10 years	22 (32%)	0.008
	≥10 years	46 (68%)	
Weight ^2^	<4 kg	39 (57%)	0.225
	≥4 kg	29 (43%)	
Neutered	Yes	54 (79%)	<0.001
	No	14 (21%)	

^1^ Mean age: 11.6 years ± 4.3 (range: 2–22); ^2^ mean body weight: 4 kg ± 1.7 (range: 1.6–8.5).

**Table 2:** Ultrasound findings of the left and right adrenals in clinically ill cats showing adrenomegaly (either uni or bilateral).

Characteristics	Classification	Left Adrenal	Right Adrenal	Left Adrenal versus Right Adrenal (*p*-Value)
Dimension (mm) ^1^	-	5.5 ± 0.8 (4.0–7.9)	5.1 ± 0.9 (3.1–7.8)	0.005
Limits	Regular	52 (81%)	55 (86%)	0.629
	Irregular	12 (19%)	9 (14%)	
Shape	Oval	41 (64%)	20 (31%)	0.005
	Bipolar	17 (27%)	26 (41%)	
	Fusiform	6 (9%)	18 (28%)	
Cortico-medullary differentiation	Without	50 (78%)	54 (84%)	0.344
	With	14 (22%)	10 (16%)	
Echogenicity	Hypoechogenic	26 (41%)	33 (52%)	0.186
	Mixed	31 (48%)	25 (39%)	
	Hyperechogenic	7 (11%)	6 (9%)	
Echotexture	Homogeneous	40 (63%)	42 (66%)	0.804
	Heterogeneous	24 (38%)	22 (34%)	
Hyperechogenic foci	Present	9 (14%)	5 (8%)	0.289
	Absent	55 (86%)	59 (92%)	
Focal lesions	Present	3 (5%)	1 (2%)	0.500
	Absent	61 (95%)	63 (98%)	

^1^ Dimension is presented as mean ± SD (minimum, maximum). Dimension values of each adrenal gland were calculated taking into account the total number of cats classified with adrenal enlargement. As some cats only had unilateral enlargement, the correspondent contra-lateral was normal, reason why the minimum range values presented are below the used cut-off of adrenal enlargement.

## Data Availability

Not applicable.
